# Verification of a neuromorphic computing network simulator using experimental traffic data

**DOI:** 10.3389/fnins.2022.958343

**Published:** 2022-08-08

**Authors:** Robert Kleijnen, Markus Robens, Michael Schiek, Stefan van Waasen

**Affiliations:** ^1^Central Institute of Engineering, Electronics and Analytics–Electronic Systems (ZEA-2), Forschungszentrum Jülich GmbH, Julich, Germany; ^2^Faculty of Engineering, Communication Systems, University of Duisburg-Essen, Duisburg, Germany

**Keywords:** neuromorphic computing, neuromorphic platform, network simulator, communication network, simulator verification, SpiNNaker, neuron mapping

## Abstract

Simulations are a powerful tool to explore the design space of hardware systems, offering the flexibility to analyze different designs by simply changing parameters within the simulator setup. A precondition for the effectiveness of this methodology is that the simulation results accurately represent the real system. In a previous study, we introduced a simulator specifically designed to estimate the network load and latency to be observed on the connections in neuromorphic computing (NC) systems. The simulator was shown to be especially valuable in the case of large scale heterogeneous neural networks (NNs). In this work, we compare the network load measured on a SpiNNaker board running a NN in different configurations reported in the literature to the results obtained with our simulator running the same configurations. The simulated network loads show minor differences from the values reported in the ascribed publication but fall within the margin of error, considering the generation of the test case NN based on statistics that introduced variations. Having shown that the network simulator provides representative results for this type of —biological plausible—heterogeneous NNs, it also paves the way to further use of the simulator for more complex network analyses.

## 1. Introduction

The introduction of novel neuromorphic computing (NC) platforms in recent years, offers great potential either in the field of cognitive computing applications or in computational neuroscience. In cognitive computing, the novel systems, like TrueNorth (Akopyan et al., 2015), are used for the application of so called artificial intelligence concepts to solve computation demanding tasks more efficiently than traditional von Neumann systems. In computational neuroscience, the focus is on the efficient simulation of large scale spiking neural networks (SNN) using novel systems like BrainScaleS (Schemmel et al., 2010), Neurogrid (Benjamin et al., 2014), or SpiNNaker (Furber et al., 2014). All of these systems are built on the concept of high parallelism, just as can be found in SNNs. Another principle relates to biological communication where the information is coded in the precise spike time. In the NC systems, this information is collected in the form of spike packets. Here, information is coded in the time that a spike happens and not in the actual bits themselves. Hence, the communication network responsible for the transfer of the spike packets is a critical component, especially in biological representative SNN with a high level of connectivity. While current systems are an improvement over traditional von-Neumann computers for the simulation of SNN, further improvements can still be made in regards to scale and acceleration.

In order to better understand the performance of communication networks and communication protocols in these systems in an early design phase, we developed a Python-based network simulator introduced in a previous work (Kleijnen et al., 2022). With the help of the simulator, the communication network load can be estimated at a high level of abstraction during the conception phase of the design. This way, different concepts can be evaluated with little effort without the need to create specialized hardware, offering great flexibility to the designer. It should be mentioned that other network simulators do exist, such as the one introduced in Ghasempour et al. (2015). This particular simulator offers a higher level of detail simulating the SpiNNaker interconnect network, but offers less flexibility for other concepts in general. However, in this paper, we focus on the comparison of our simulator with experimental results presented in the literature.

An important precondition for the effectiveness of this methodology is that the simulator is able to accurately represent the performance of a physical hardware system. This paper focuses on the comparison between the network simulator introduced in one of our previous works and the network traffic measured on an actual NC system, namely the SpiNNaker system. This comparison is performed for three different types of neuron mappings in order to cover a range of scenarios. This way, it can be confirmed that the simulator represents the network load on a real system correctly and can be used as a first stage design tool reliably. First, in Section 2.1, a brief overview of the Python-based network simulator is given. Then, in Section 2.2, SpiNNaker—the NC hardware system used for comparison—is described, with emphasis on the communication network. Section 2.3 discusses the experiments used as benchmark in this work and in Section 2.4, the corresponding settings for the simulator to mimic these experiments are outlined. In Section 3, the results of the simulations are presented, discussed, and compared to the experimental data. Finally, in Section 4, the summary and conclusion are given, along with a discussion regarding potential improvements and future work.

## 2. Materials and methods

### 2.1. NC communication network simulator

In a previous study, we introduced an in-house implemented network simulator specifically designed to evaluate network load and latency on an NC communication network. In this subsection, we will briefly describe this simulator, a more detailed description can be found in Kleijnen et al. (2022) and its supplementary material. The simulator operates in four steps: generation of a neural network (NN), generation of the hardware graph, assignment of neurons to computational nodes, i.e., neuron mapping, and the simulation of the spike packet movement created by each neuron. The first step creates a NN, based on biological connectivity information, which is used as a test case during the simulation. The connectivity and size of the NN can greatly impact the network load on the system. As such, it is important to generate a NN that represents the eventual use case accurately to get accurate simulation data. The second step creates a directional graph that is used to model the communication network. Vertices in the graph represent the computational nodes of the NC system while the edges represent the communication links. The graph can take any arbitrary shape, but in most hardware systems, the communication network can be represented with a regular mesh structure. The next step in the simulation combines the two objects created before and assigns the neurons to a location in the hardware graph. How the location of each neuron is determined can vary greatly and depends on multiple factors, such as hardware node constrains, e.g., the maximum number of neurons per node, as well as mapping algorithms. More sophisticated mapping algorithms can reduce the network load significantly, as is shown in Section 3, but might require a longer start up time for the system. The final step in the simulation is the determination of the communication traffic. Neurons are assumed to spike, and the route and distance that the resulting spike packet takes to reach the post-synaptic neurons are calculated. The resulting route is a list of nodes and links passed by the spike packet, while the distance is given in the number of nodes and links passed to reach its farthest destination. In reality, a neuron only fires a spike event if its membrane potential surpasses a certain threshold. However, the simulator does not model the neuron behavior, instead, it assumes that each neuron in the SNN fires once. In order to consider the effect of differences in neural activity of different neuron populations on the network load, different neurons are assigned different fire rates (FRs). These FRs are then used to weigh the contribution of each neuron to the network load. The overall network load is determined by adding the contributions of individual neurons together, weighted by their respective FR. The network load is then reported as the number of spikes going over each link, and the number of internal and external packets crossing through each node. External packets are defined as packets originating from neurons not located in the local node, while internal packets originate from local neurons. In addition, network latency is reported as the maximum distance a generated spike packet has to travel for each individual source neuron. If timing values to pass a node or link are available, these values can be used as a multiplier in order to estimate the system's latency in seconds. However, in the early design phases, when these variables are still unknown, a useful alternative is to set *t*_*node*_ = 1 and *t*_*link*_ = 0. This results in a latency estimation in the number of “hops” that a spike has to travel.

### 2.2. The SpiNNaker system

Multiple different NC simulation platforms have been developed in recent years (Schemmel et al., 2010; Benjamin et al., 2014; Furber et al., 2014; Akopyan et al., 2015; Furber, 2016; Thakur et al., 2018; Young et al., 2019). These systems do share some similarities with each other, as they are all inspired by the same fundamental principles found in the biological brain. Nonetheless, large differences between their design philosophies are also apparent. Especially their field of application has a major influence on the design requirements and consequently the design concept. In favor of detailed communication traffic results reported in Urgese et al. (2015, 2018) that allow for a comparison with results obtained by our simulator, we limit our analysis to the comparison between our simulator and experimental data found using the SpiNNaker system. Because of this, we will only describe the SpiNNaker design in more detail. A general overview of the other different systems and more detailed descriptions of some of the individual systems can be found in Schemmel et al. (2010); Benjamin et al. (2014); Furber et al. (2014); Akopyan et al. (2015); Furber (2016); Young et al. (2019), and Thakur et al. (2018), respectively.

The SpiNNaker system is designed to model large-scale SNNs in biological real time. The system is fully digital and uses a large number of chips to achieve the level of parallelism desired. The individual chips operate synchronously but communicate with each other in an asynchronous manner to reduce energy consumption, resulting in a globally asynchronous—locally synchronous system. The foundation of the system is the SpiNNaker chip. Each SpiNNaker chip contains 18 ARM968-cores, a custom router, system ROM, system controller, system RAM, external SDRAM, and an Ethernet interface. Each of the 18 ARM-cores has its own DMA controller and two tightly coupled memories for instructions and data. The cores are connected to the other elements by a system Network-on-Chip.

During the start-up phase of the system, each chip tests the cores functionality and picks one core to operate as a monitoring core which coordinates chip-level functions, such as non-spike communications, control, and management. Sixteen of the remaining cores will be responsible for the modeling of neurons. The eighteenth core is kept in reserve to increase production fault tolerance and extend its lifetime. The neural modeling in the cores is performed on a software level, resulting in a great level of flexibility regarding neuron models and parameters, and simulation parameters such as time step size. Depending on the complexity of the neuron model used for the neurons modeled by a specific core—a specific core can only model neurons with the same neuron model—and the time resolution chosen, the maximum number of neurons per core (NpC) can vary between 80 and 1,000. Once a spike event happens, the core creates a spike packet which is communicated within the chip and/or system *via* the on chips router.

**Figure 1:**
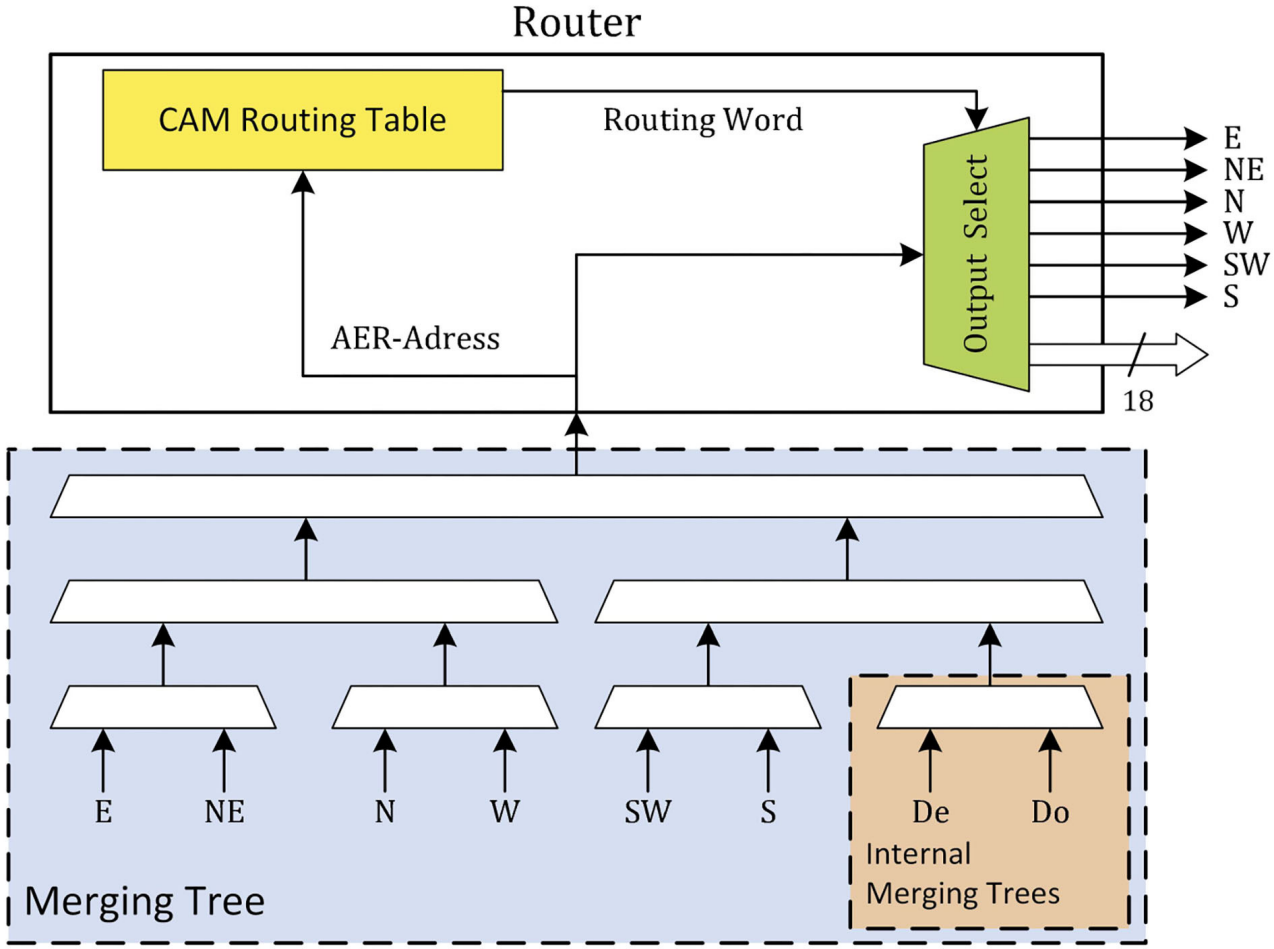
Role of merging trees in the SpiNNaker router.

The router is the basic building block of the communication network on the SpiNNaker system. Each router is connected to the chips' internal resources, the local cores, and the routers of the six neighboring chips. These six neighbors are identified as north (N) and south (S) (vertical neighbors), east (E) and west (W) (horizontal neighbors), and north-east (NE) and south-west (SW) (diagonal neighbors) and form a triangular mesh network. The local cores communicate to the router *via* two merging trees. These two merging trees (De and Do) are then merged together with the other six input ports (N, NE, E, S, SW, and W) and passed to the router as shown in Figure 1. Four different types of packets are used in the system: nearest-neighbor (NN), point-to-point, multicast (MC), and fixed-route. Each of these packet types has its own application, with the MC type being the most relevant during the simulation of the SNN. MC packets are used to distribute the spike information through the system to the targeted chips and cores. This is done by use of an address event representation (AER) protocol. The payload of the MC packet only contains a unique source neuron identifier, the rest of the spike information is encoded in its timing. Once a router receives an MC packet, it looks up the source neurons ID in a ternary content addressable memory (TCAM). Each entry key in this TCAM returns a 24-bit word which consists of a 6-bit mask as an external link indicator and an 18-bit mask as an internal core address. Every “1” entry in this bit word marks an output port of the router to which the packet needs to be transmitted. For example, “010011 0001100101100010” translates to the router sending the packet further through external ports NE, SW, and W (counts counter-clockwise starting from the east port) and to local cores 3, 4, 7, 9, 10, and 14. This way, packets are duplicated along the way in an effort to reduce traffic as only a single packet is sent over the initial part of the route. To reduce the size of the TCAM, the number of entries is reduced through the use of a default route. In case the router cannot find a match for the source neuron ID, the packet is forwarded to the output port opposite of the port it came from. This way, TCAM entries are only needed in the nodes where the spike packet needs to take a turn or has to be delivered to one or more of the local cores. Efficient mapping and routing algorithms will use the number of TCAM entries required as an additional constraint during the mapping and routing phase. This and a number of other techniques (Mundy et al., 2016) are used by the SpiNNaker toolchain in order to minimize the number of TCAM entries required, i.e., keep the number of TCAM entries required below the available TCAM size.

### 2.3. Experimental setup

In Kleijnen et al. (2022), the performance of our simulator was validated against a numerical model (Vainbrand and Ginosar, 2011) and put into relation to an analytical model presented in Kauth et al. (2020). However, both these models are limited to the use of homogeneous connectivity models. To validate the simulators performance for—biological representative—heterogeneous connectivity models, a different approach has to be used. The best way to validate the correctness of the simulator is to run a network load analysis for a certain NN using the simulator and compare the results to empirical data measured, running the same NN on actual NC hardware. Since convenient results obtained from a hardware system were already reported in Urgese et al. (2015, 2018), we compare our simulation results against experimental data found in these literature sources. They cover an investigation of a bottleneck of the SpiNNaker communication infrastructure and different neuron mapping procedures. The first aspect was investigated by passing a large number of spike packets through a single node and measuring the number of spikes dropped, i.e., not reaching their destination, for different test configurations. The conclusion from this analysis was that more packets are dropped when a port of the SpiNNaker chip has to communicate in both directions. Unfortunately, we cannot recreate this aspect using our simulator, as the simulator is not time based and does not take timing delays and input-/output- buffers into consideration.

On the other hand, one unique feature of our simulator is its ability to evaluate the network load for different neuron mapping algorithms. As such, we can attempt to recreate the results obtained from the respective experiments.

Such experiments were performed using a scaled down version of the cortical microcircuit model (Potjans and Diesmann, 2014; van Albada et al., 2018) as the test case. This model consists of four layers each with two populations, an excitatory and an inhibitory population. Additionally, in Potjans and Diesmann (2014), the model also contains a “thalamic” population, which is omitted in van Albada et al. (2018) and Urgese et al. (2015, 2018). Each population has its own specific size, i.e., number of neurons, as well as a defined connection probability to neurons from other populations. To scale down the network, the number of neurons in each population was reduced to 5% (N05) and the number of synapses per neuron was reduced to 20% (K20) while conventionally the number of neurons and the number of synapses per neuron are scaled by the same fraction. On the SpiNNaker system, each neuron was modeled as an integrate and fire (IF) neuron and was set up with specific parameters corresponding to the neuron model of the specific population.

In addition to the scaling, some alterations were made to the NN in order to run it on the hardware and match the temporal behavior of the model. The connectivity of the model remained the same, but due to hardware restrictions, some elements needed to be added. The first additional element type are spike source (SRC) neurons. They simulate background activity originating from areas of the brain not included in the model as spike trains generated with a Poisson probabilistic process. These spike trains form the majority of all spikes in the system, a characteristic that is important for the mapping as well. More details will be provided later in this section. Because of the large number of spikes, it is infeasible to send them to the corresponding SpiNNaker chips from an external host. Instead, the spike trains are generated by the SRC neurons, where SRC neuron is associated with one IF neuron and mapped to cores within the SpiNNaker chips themselves. This way, only the parameters of the desired Poisson distribution have to be loaded and the spike trains can be generated on the fly, in the system itself. The second addition was the use of delay extension (DE) neurons. The cores assigned to model the IF neurons are able to handle synaptic delays up to 16 time steps. However, with a simulation time step of 0.1 ms and normally distributed delays with a 1.5 ms mean value for excitatory neurons, some delays will be larger than the available 1.6 ms. To create delays greater than 16 time steps, the DE neurons were used as described in van Albada et al. (2018). Due to their simple neuron model—these neurons fire 1-to-1 in response to incoming spikes with a specified delay—these neurons are not limited to the same maximum number of NpC as the IF and SRC neurons are.

**Figure 2:**
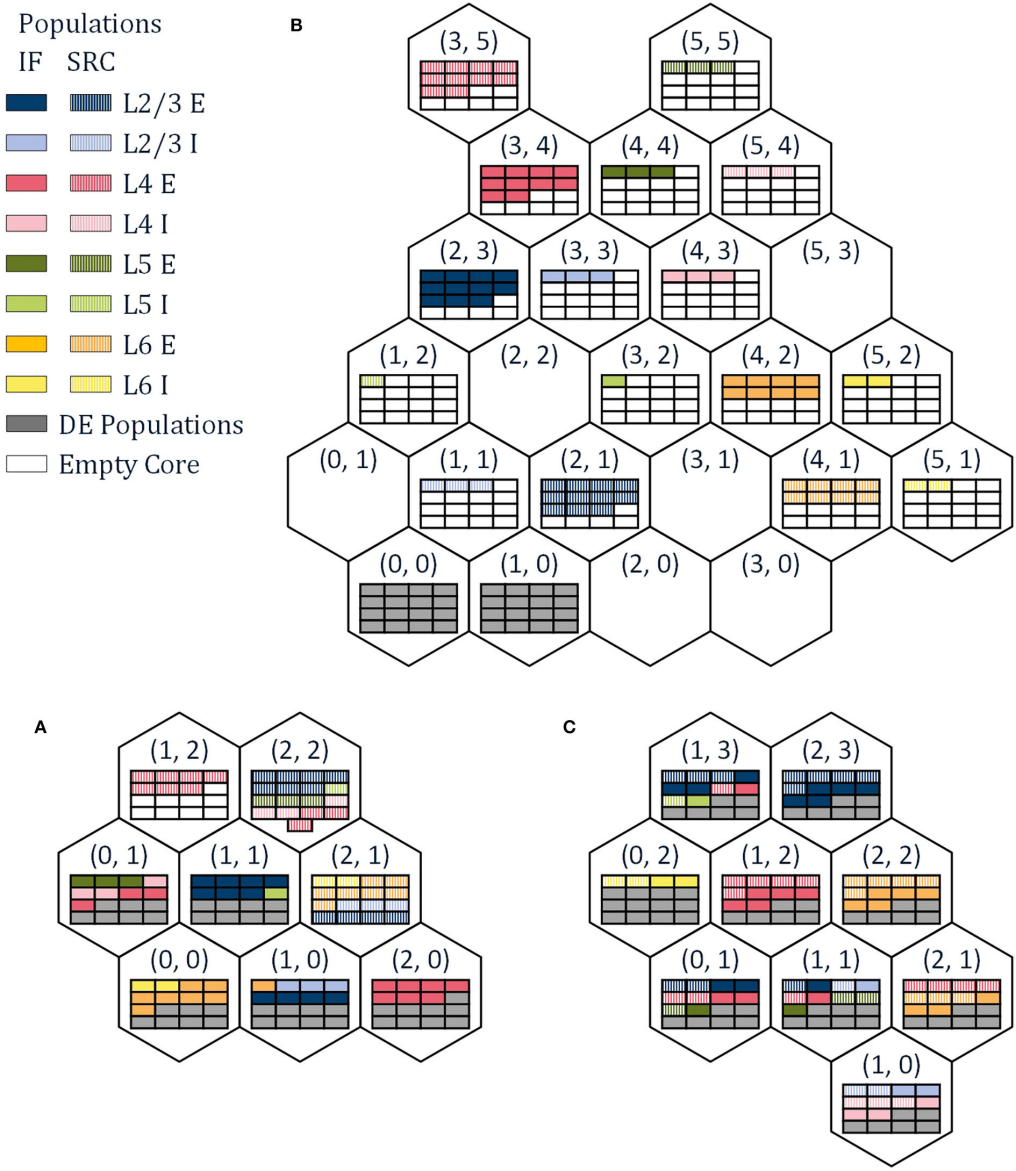
The resulting neuron maps for the three different mapping approaches used in this work, **(A)** PArtition and Configuration MANager (PACMAN), **(B)** MANUAL, and **(C)** GHOST.

The reference papers (Urgese et al., 2015, 2018) investigate three different mapping procedures, PACMAN, MANUAL, and GHOST. A number of alternative mapping approaches are described in Heathcote (2016) and Pettersson (2021) but will not be discussed in this work. PArtition and Configuration MANager (PACMAN), as presented in Rhodes et al. (2018); Rowley et al. (2019) for example, is the native python package used to configure the SpiNNaker boards to run a given SNN according to a PyNN SNN description. This procedure divides each population into part-populations. Each part-population contains the maximum number of neurons possible to place per core with exception of the last part-population which contains the remaining neurons. Then, the part-populations are assigned to cores of a SpiNNaker chip sequentially. For every IF part-population assigned to a chip, spaces are reserved for the corresponding DE populations. Once all cores of a chip have been filled, including the cores reserved for DE neurons, PACMAN progresses to the next chip. The order of the chips is determined by the radial distance around a chip of choice (Barchi et al., 2018) in this case chip (0, 0). The resulting mapping solution is shown in Figure 2A. Due to the simplicity of the algorithm, it requires only a minimal amount of computational power and can be used for large SNN as well, as is shown for the full scale cortical microcircuit model in Rhodes et al. (2020). However, this comes with its own limitations. The maximum number of NpC is the only constraint while splitting the populations, and it does not consider any network connectivity while placing the individual part-populations. This potentially leads to very small part-populations and large distances in the hardware between highly connected part-populations.

The second mapping procedure is a MANUAL approach, with the corresponding mapping solution shown in Figure 2B. This mapping solution was hand-picked in an attempt to achieve mono-directionality in the communication between SRC populations and IF populations. While this procedure did reduce the number of external packets by 33% and the number of dropped packets—the aspect not considered in this work—to zero, it is still far from optimized. For this mapping procedure, the number of spikes generated per population can be read out as the number of internal spikes in the corresponding node, as each node only simulates a single population. If we compare the number of spikes generated by the SRC populations and the IF populations, we can see a significant difference between the two. The source neurons will create the majority of spikes in the system—98%—even though they only have to travel a relatively short distance, to a single destination. With this mapping procedure, all the DE neurons are assigned to the nodes (0, 0) and (1, 0).

To reduce the traffic through the network further, it would be beneficial to prevent inter-chip communication from SRC neurons to IF neurons. This can be achieved by assigning SRC neurons to the same chips as the corresponding IF neurons. This way, the spikes generated by the SRC populations, only have to be transported within the node and do not contribute to the number of external packets per node. The same idea can be applied to the DE neurons as well, while also considering the connectivity between part-populations to determine the location of the IF neurons. The third mapping procedure introduced in Urgese et al. (2018), GHOST mapping, does exactly this. By iteratively sub-clustering the groups of neurons from the same population and cluster, the neurons are divided into part-populations that comply with the maximum number of NpC. Then, the Sammon mapping algorithm is used to map the part-populations to the individual chips. To ensure that the SRC- and DE-neurons are mapped onto the same chips as their corresponding IF neurons, cores on each chip are reserved for the SRC- and DE- part-populations and assigned to correct SRC populations at the end of the placement procedure. The resulting neuron mapping is shown in Figure 2C. It should be noted that in Knight and Furber (2016) and Peres and Rhodes (2022) a technique is presented which removes the traffic created by the SRC neurons entirely from the interconnect network, allowing the simulation of full-scale models in real time. However, as this technique is not used in the experimental setup, it will not be discussed in detail in this paper.

### 2.4. Simulation setup

To validate the correctness of our simulator, we recreate the network load measured with the previously described SpiNNaker test setup, by running the simulator with a comparable set up. We represent the SpiNNaker communication network as a triangular mesh and set the casting type to MC. As a test case, we use the scaled down cortical microcircuit model, or its connectivity information, respectively, including the additional SRC and DE neurons. This SNN will not be exactly the same as the SNN used in the experiment, but it will be statistically representative. In the NC hardware, traffic is only counted as external traffic (router-to-router—R2R) if it originates from outside the SpiNNaker chip, communication generated locally, between cores and the router is counted as internal traffic (core-to-router—C2R). The same classification is applied by the simulator while representing the SpiNNaker chips as the nodes of the network. As the simulator does not distinguish between the different cores on a chip and combines them into one node, the variable *NpN* is set to 16×*NpC*, but exceptions are made when assigning DE neurons, due to their simpler neuron models.

As the simulator does not try to simulate neuron dynamics, the neural activity has to be defined by setting the FRs of the neurons. Urgese et al. (2015) offers us the possibility to calculate the average firing rates of each population by dividing the number of internal spikes in each node, by the number of neurons of the population assigned to that node. Unfortunately, we cannot do this for the DE populations as there are multiple populations combined within the nodes. Because we know that these populations simply delay the spikes of the original IF neurons, we assume the same FR for these populations as for their corresponding IF populations. In Urgese et al. (2015), these values do not exactly match the number of internal spikes of the IF neurons, which will most likely be caused by spikes falling outside the measurement time-window due to the delays. Yet, the experimental data are approximated relatively well as will be seen below. The average firing rates for the populations are given in Table 1.

**Table 1:** Average firing rates of the different populations of the spiking neural network (SNN) test case.

**Populations**	**SRC neurons**	**IF and DE neurons**
L2/3E	2560.78	0.8491
L2/3I	2422.62	3.5979
L4E	3362.28	3.8904
L4I	3041.53	7.0293
L5E	3195.97	8.4298
L5I	3048.68	9.2453
L6E	4635.81	1.1516
L6I	3353.83	8.5986

**Figure 3:**
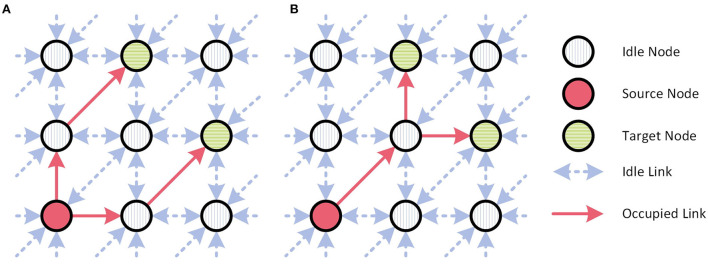
The difference in network load between two possible shortest routes to reach all destinations with an MC packet, **(A)** 4 different links used and **(B)** 3 different links used.

Finally, the experiments were performed using the neighbor exploring routing (NER) algorithm. This algorithm calculates the shortest route to the destination, from the closest, previously visited node—including the source node. This routing algorithm was also implemented in the simulator and thus will be used for the comparison. However, the routes calculated by this algorithm vary depending on the implementation of the algorithm. Additionally, a couple of factors, such as the routing order, the maximum search range, routing table entry constraints, and branch node constraints affect the resulting routes. A more detailed explanation of the algorithm, including the effects of the different factors listed above is given in Navaridas et al. (2015). In this study, the maximum number of routing table entries and limitations regarding the maximum search range and which nodes can be branch nodes were neglected and the destinations were sorted in order from closest to furthest from the source node. Nonetheless, differences might still occur as it's not clear how these constraints were handled in the experimental setup. The resulting variations may be reflected in both the distribution of traffic and the total number of external packets. In a regular mesh, there are multiple different “shortest” paths to a destination. The route actually chosen determines which nodes will count the external packet. However, even if the route returned by the algorithm has the shortest possible length from the source to the destinations, the total number of links occupied by a combined MC packet can still vary as shown in Figure 3.

## 3. Results

In this section, we present and evaluate the results obtained with the simulator setup described in Section 2.4. With respect to these results, it is important to mention the difference between the numerical data returned by the simulator and measured on the hardware platform. In the SpiNNaker system—or any physical NC system for that matter—the number of packets going through the routers will always be positive integers. The simulator on the other hand, accepts any arbitrary (positive) real numbers as FRs of the neurons, which are used as multipliers for the neurons' generated network load. Thus, the resulting number of spikes per link and router can also be any arbitrary real number. The results of the different simulations are shown in Tables 2–4. They are rounded to a single decimal place. The total values, however, are calculated using the non-rounded values, so that there may be a small discrepancy between the total values stated and a simple column sum. Tables 2, 3 also include the results presented in Urgese et al. (2015) identified as “Experimental.”

**Table 2:** Simulated communication traffic results for the test case mapped with the MANUAL procedure.

	**External packets**		**Internal packets**		**Neurons**	
**Node**	**Simulated**	**Experimental**	**Simulated**	**Experimental**	**(SRC and IF)**	**Description**
(0, 0)	3 456.0	3 456	3 456.0	4 351	0	DE Populations
(0, 1)	0	3 146	0	0	0	Not Used
(1, 0)	11 679.0	8 232	9 270.0	9 129	0	DE Populations
(1, 1)	4 503.0	17 740	704 983.0	704 983	291	SRC_L2/3I
(1, 2)	0	18 001	161 580.0	161 580	53	SRC_L5I
(2, 0)	1 264.0	2 092	0	0	0	Not Used
(2, 1)	19 685.0	15 040	2 647 850.0	2 647 850	1034	SRC_L2/3E
(2, 2)	3 527 800.0	3 532 713	0	0	0	Not Used
(2, 3)	2 669 406.0	2 675 945	878.0	878	1034	L2/3E
(3, 0)	0	1 264	0	0	0	Not Used
(3, 1)	1 264.0	828	0	0	0	Not Used
(3, 2)	185 278.0	191 753	490.0	490	53	L5I
(3, 3)	726 860.0	727 444	1 047.0	1 047	291	L2/3I
(3, 4)	3 700 361.0	3 706 900	4 260.0	4 260	1095	L4E
(3, 5)	0	0	3 681 697.0	3 681 697	1095	SRC_L4E
(4, 1)	0	1 264	3 333 150.0	3 333 150	719	SRC_L6E
(4, 2)	3 357 774.0	3 364 249	828.0	828	719	L6E
(4, 3)	850 363.0	856 929	1 919.0	1 919	273	L4I
(4, 4)	794 308.0	800 847	2 040.0	2 040	242	L5E
(5, 1)	0	0	493 013.0	493 013	147	SRC_L6I
(5, 2)	517 201.0	520 765	1 264.0	1 264	147	L6I
(5, 3)	0	10 144	0	0	0	Not Used
(5, 4)	0	6 300	830 338.0	830 338	273	SRC_L4I
(5, 5)	0	0	773 424.0	773 424	242	SRC_L5E
**Total**	16 371 202.0	16 465 052	12 651 487.0	12 652 241	7708	

**Table 3:** Simulated communication traffic results for the test case mapped with the PACMAN procedure.

	**External packets**		**Internal packets**		**Neurons**	
**Node**	**Simulated**	**Experimental**	**Simulated**	**Experimental**	**(SRC and IF)**	**Description**
(0, 0)	3 759 394.3	3 773 911	4 140.2	4 882	847	2 Populations
(0, 1)	2 625 117.9	2 630 277	10 252.2	12 729	815	3 Populations
(1, 0)	4 512 765.8	5 587 441	2 817.1	3 334	710	3 Populations
(1, 1)	8 217 079.4	4 424 623	2 056.7	2 412	687	2 Populations
(1, 2)	2 612 446.1	0	2 673 012.9	2 672 252	795	SRC_L4E
(2, 0)	2 689 547.7	2 692 613	6 185.8	9 471	795	L4E
(2, 1)	221.9	2 696 375	5 555 459.3	5 559 023	1,557	4 Populations
(2, 2)	0	2 672 252	4 397 562.8	4 397 319	1,502	5 Populations
**Total**	24 416 573.1	24 477 492	12 651 487.0	12 661 422	7708	

**Table 4:** Simulated communication traffic results for the test case mapped with the GHOST procedure.

**Node**	**External packets**		**Internal packets**	**Neurons**	**Description**
	**Simulated**	**Experimental**	**Experimental**	**(SRC and IF)**	
(0, 1)	19 707.6		1 409 414.8	934	6 Populations
(0, 2)	22 924.0		495 541.0	294	2 Populations
(1, 0)	16 710.0		1 305 560.7	934	4 Populations
(1, 1)	18 564.8		1 330 679.5	904	8 Populations
(1, 2)	21 561.6		1 685 030.6	1,000	2 Populations
(1, 3)	20 694.8		1 218 815.9	868	6 Populations
(2, 1)	23 291.8		1 914 925.5	934	4 Populations
(2, 2)	24 415.6		2 087 152.6	900	2 Populations
(2, 3)	21 552.6		1 204 366.4	940	2 Populations
**Total**	189 422.8	250 000	12 651 487.0	11,562	

We start off by discussing the results obtained using the MANUAL approach, deviating from the original order of mapping procedures given before. This is done, as this approach isolates all populations to individual nodes which gives a clearer overview of what is happening in the system. For this algorithm, we can see that the number of internal packets per node, matches almost perfectly with the experimental data. This was to be expected, as this data was used to set up the average firing rates. However, we can also see the difference in nodes (0, 0) and (1, 0) due to the DEs as discussed before. Overall, the neural activity is well represented by the used FR values and as a result, the data for the total number of external packets in the system matches with experimental data as well. The number of external packets for the individual nodes, on the other hand, shows some differences. This can be attributed to the dependence on the actual implementation of the NER algorithm and the resulting routes as explained in Section 2.4.

‘For the PACMAN algorithm, the difference between the simulated total number of internal packets and the experimental data is slightly larger. As we used the same FR values for both simulation runs, the number of generated spikes should be identical, which we can observe from the simulation results. However, in the experimental data, the total number of generated spikes is larger. Unfortunately, we are unable to extract more accurate FR values from the experimental data of this run as multiple populations are placed on individual nodes. This difference might also be enlarged by the reinjection mechanisms of the SpiNNaker system which retransmit packets that are lost. The difference is relatively small for the total values of packets but is especially apparent from the nodes containing the IF and DE neurons. Because the spike packets originating from these neurons generally have to go to multiple nodes at a larger distance, the reduction of internal packets will reflect in the number of external packets in an amplified way, as can also be seen in Table 3. The distribution of these external packets over the different nodes again depends on the actual implementation of the routing algorithm, and as such differs significantly. However, the difference in the total number of external packets is still acceptable considering the different sources of variation which were discussed in Section 2.4.

The simulation results for the GHOST mapping algorithm are shown in Table 4. Unfortunately, for this algorithm, only a total of 250,000 external packets is reported for the experimental data. The difference with the simulated total number of 189,422.8 external packets is substantially larger for this setup. Nonetheless, the simulated traffic is of the same order of magnitude as the experimental data, which is largely reduced in comparison with the other two experiments. Thus, the simulator did manage to capture this reduction of the network load due to the improved mapping procedure.

## 4. Discussion

### 4.1. Summary

**Figure 4:**
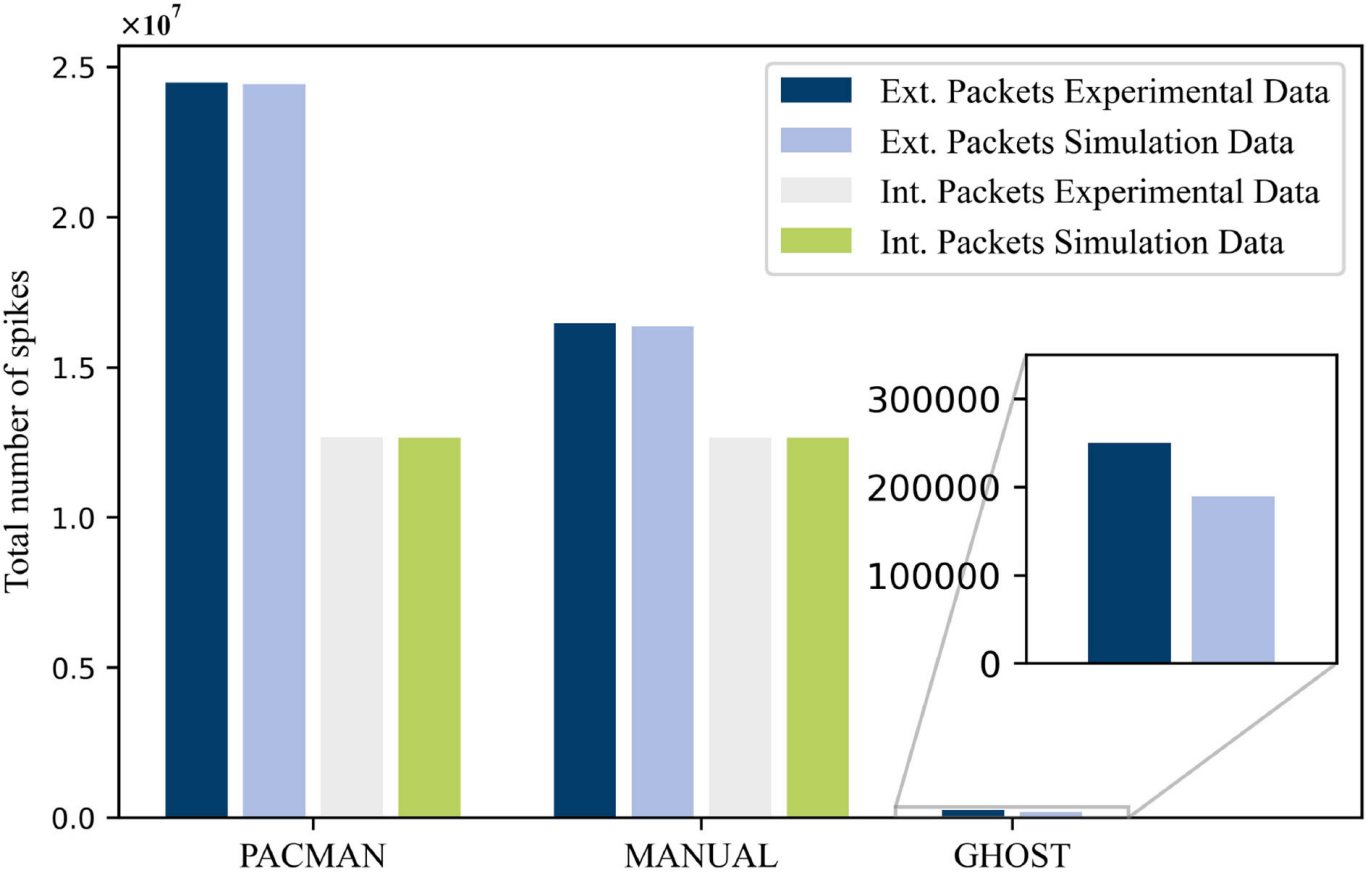
The total number of spike packets measured in the experimental setup compared with the simulated values for the different mapping procedures.

In this paper, we presented simulation results obtained with an in-house developed network simulator. These simulations were performed in an attempt to reproduce experimental data measured on the SpiNNaker system. This was done for three different types of neuron mapping procedures: PACMAN, MANUAL, and GHOST. The total number of external spikes and internal spikes, where applicable, measured for these three procedures is shown in Figure 4. For both the PACMAN and the MANUAL mapping procedure, the simulated number of external packets in the system corresponds very well to the experimental data. The small differences observed for these two scenarios, 0.25 and 0.5%, respectively, are well within the margin of error considering the different potential causes for variations. For the last mapping procedure, a substantially larger difference of 24.2% was observed between the simulator and the experimental data. Unfortunately, we cannot identify the exact causes of this larger variation due to the lack of a detailed overview of the experimental network load. Nonetheless, the simulator did capture the same level of reduction of external packets caused by the GHOST mapping approach. As the simulator is meant as a first stage design tool, it will most likely be used for qualitative comparisons and this will be sufficient. This especially applies, because the exact parameters of the test case, such as the FR of the different populations, are still unknown at this point. Other factors like the connectivity or even the entire test case, and with it the mapping, might also change for different analyses performed using the NC hardware.

### 4.2. Discussion

There are a couple of potential reasons which can explain the larger variation for the GHOST mapping experiment. As discussed before, the FR values of the IF and DE neurons extracted from the MANUAL experiment do not necessarily represent the FR values of the other experiments correctly. For the other experiments, i.e., in case of the PACMAN or the MANUAL algorithm, the total number of external packets was dominated by packets originating from the SRC neurons, which are represented well by the FR values. Because of this, the relative difference was neglectable previously. In case of the GHOST algorithm, however, the SRC neurons are located on the same nodes as the corresponding IF neurons and have no impact on the number of external packets. Thus, while the absolute difference is almost comparable for all experiments, approximately 60,000 external packets, the relative difference is significantly higher. A second potential cause for the observed difference are variations between the neuron activities within a single population. The simulator assumes an average firing rate for all neurons in a population. In an actual SNN, on the other hand, different neurons within a population can and will have different rates of activities. With all the neurons of one population placed onto a single or maximum 2 nodes, which was the case for the first two experiments, these variations will average out. In the case of the GHOST algorithm, however, the populations are spread out more and the variations can have an impact. This may be especially the case when highly interconnected neurons that potentially reveal a shared lower or higher activity due to their high connectivity are concentrated into the same subgroup. A final potential cause is an obscurity in the mapping solution used in Urgese et al. (2018). The paper gives a detailed explanation of algorithm's operating principle, however, the resulting mapping solution is only visualized in an abstract manner. This is sufficient to derive a comparable neuron mapping, but not the exact same neuron placement, especially in combination with an SNN which is only statistically equal. As most of these potential causes are a result of not having detailed enough input data, there are possibilities to improve the comparison between experiments and simulations by having access to more detailed experimental parameters. Ideally we would perform both the experiment and the simulations ourselves, so the exact same NN can be used for both. This way, the exact FR of each individual neuron can also be determined, just as the exact neuron mapping and the routing algorithm. Another way to improve confidence is to compare a larger number of different scenarios. The results between simulation and experiment might not match perfectly for every scenario, but the same relative behavior might be observed between the two.

Another point of discussion is the metric used by the simulator. To estimate the network's performance, the simulator returns a latency value and the number of spike packets passing through each link and node. However, in the SpiNNaker system, the interconnect performance is generally measured by the number of packets being dropped. Due to the high level of abstraction of the simulator, it is not possible to simulate this. The probability of packets dropping can be estimated by comparing the maximum throughput simulated and the maximum number of packets the routers can handle. If the former is larger than the latter, packets will be dropped, but even if the throughput is slightly below the maximum capacity, the probability of packets being dropped is relatively high. Often, even the probability of dropping a packet can be seen as bad. However, as described in Urgese et al. (2015, 2018), the maximum number of packets a router can handle is not constant and depends on the directions of the different incoming and outgoing traffic streams, making it difficult to accurately predict the occurrence of dropped packets.

### 4.3. Outlook

With the behavior of the simulator being verified against the performance of actual hardware, the simulation results can be used during the design phases with confidence. A first step will be a design-space exploration in order to find the strengths and weaknesses of certain general designs. As a next step, we will come up with new communication concepts and evaluate their performance. If a concept proves to be advantageous, a more in depth analysis will be performed and potentially implemented in hardware.

## Data availability statement

The simulation scripts and source codes used in this work to demonstrate correctness are available online at: https://github.com/Rkleijnen/NeuCoNS (https://www.doi.org/10.5281/zenodo.6862974).

## Author contributions

RK contributed to the conception and design of the study and wrote the first draft of the manuscript. MR, MS, and SW wrote sections of the manuscript. All authors contributed to manuscript revision, read, and approved the submitted version.

## Funding

This project was funded by the Helmholtz Association Initiative and Networking Fund under project number SO-092 (Advanced Computing Architectures, ACA).

## Conflict of interest

Authors RK, MR, MS, and SW were employed by the Forschungszentrum Jülich GmbH.

## Publisher's note

All claims expressed in this article are solely those of the authors and do not necessarily represent those of their affiliated organizations, or those of the publisher, the editors and the reviewers. Any product that may be evaluated in this article, or claim that may be made by its manufacturer, is not guaranteed or endorsed by the publisher.
